# Persistent Olfactory and Taste Dysfunction after COVID-19

**DOI:** 10.3390/life14030317

**Published:** 2024-02-28

**Authors:** Malgorzata Buksinska, Piotr Henryk Skarzynski, Danuta Raj-Koziak, Elzbieta Gos, Malgorzata Talarek

**Affiliations:** 1 Otorhinolaryngosurgery Clinic, World Hearing Center, Institute of Physiology and Pathology of Hearing, 05-830 Kajetany, Poland; m.buksinska@ifps.org.pl; 2 Department of Teleaudiology and Screening, World Hearing Center, Institute of Physiology and Pathology of Hearing, 05-830 Kajetany, Poland; e.gos@ifps.org.pl (E.G.); m.talarek@ifps.org.pl (M.T.); 3 Heart Failure and Cardiac Rehabilitation Department, Faculty of Medicine, Medical University of Warsaw, 03-242 Warsaw, Poland; 4 Institute of Sensory Organs, 05-830 Kajetany, Poland; 5 Tinnitus Department, World Hearing Center, Institute of Physiology and Pathology of Hearing, 05-830 Kajetany, Poland; d.koziak@ifps.org.pl

**Keywords:** anosmia, COVID-19, hyposomia, smell, phantosomia, olfaction

## Abstract

(1) Background: One of the possible symptoms of COVID-19 is a sudden loss of smell and taste. The main aim of the study was to evaluate the severity of post-COVID-19 olfactory dysfunction (OD). A secondary aim was to assess the relationship between OD and gustatory (taste) dysfunction (GD). Margins: 2.5 cm (1 inch) at top, bottom, right, and left. (2) Methods: The study group consisted of 81 subjects (16 men and 65 women) aged between 12 and 73 years. All of the patients presented to a center for subjective OD associated with COVID-19. They were tested with a Sniffin’ Sticks test (SST) for OD and a Taste Strip test (TS) for GD. (3) Anosmia was present in 18 participants (22%), hyposmia in 52 (64%), and normosmia in 11 (14%). Some 36% of the patients reported imaginary smells (phantosmia), but it did not correlate with olfactory sensitivity. Comparing the different parts of the SST showed that subjects scored lowest on the threshold part of the test. The results of the discrimination and identification parts of the test were better, implying that if the stimulus is intense enough, incorrect discrimination and identification of odors is less frequent. A sweet taste was the easiest to recognize (78% could do so), while the most difficult to recognize was salty (68%). There were weak and statistically non-significant correlations between olfactory and taste dysfunction. (4) Conclusions: The results suggest that post-COVID-19 olfactory dysfunction was more peripheral than central. Testing patients for the severity of post-COVID-19 OD may help clinicians treat the condition. Because there is no fully effective treatment, research on post-COVID-19 OD is needed.

## 1. Introduction

One of the symptoms of COVID-19, a disease caused by the SARS-CoV-2 virus, is a sudden loss of smell and/or taste. Olfactory dysfunctions (ODs) and gustatory dysfunctions (GDs) usually appear early and are sometimes the only symptoms of the disease. During the pandemic, it was postulated that a sudden loss of smell and/or taste might be the basis for a diagnosis of COVID-19 or at least used as a screening tool [1,2]. According to a meta-analysis by Agyeman and colleagues, OD is present in 41% and GD in 38% of COVID-19 patients. OD and GD are more common in the young and are not related to sex. OD in COVID-19 can be tested both quantitatively and qualitatively. The prognosis for recovery from OD in COVID-19 is good, with most patients experiencing a spontaneous return of olfactory function to pre-symptomatic levels within about a month [3]. However, in some 7–20% of patients, OD persists after other symptoms of the disease have resolved [4,5].

Patients with predominantly nasal and sinus symptoms experience swelling of the mucous membranes of the olfactory cleft and increased mucus secretion, which creates a mechanical block to odor molecules and induces conductive OD [6]. In addition, the penetration of virus particles into the non-neural cells of the olfactory epithelium triggers a massive response from the immune system. In this ‘cytokine storm’, significant amounts of pro-inflammatory cytokines, including tumor necrosis factor alpha (TNF-alpha) and interleukin 1 beta (IL-1beta), are released, damaging inflamed cells. In turn, the malfunction of non-neural cells leads to nerve cell disruption and sensory OD, in which connections between olfactory receptors and neurons conducting olfactory stimuli to the brain may be lost [7]. However, the mechanism of SARS-CoV-2 penetration into the central nervous system is not fully known. One hypothesis suggests that the virus penetrates the cerebrospinal fluid directly from non-neural cells of the olfactory epithelium and then spreads throughout the nervous system [8,9].

Better understood is the mechanism of virus entry into the olfactory neuroepithelium, which causes sensorineural OD. A spike glycoprotein (S protein) is present on the surface of the virus and is the element detected by immune cells. S protein binds to angiotensin-converting-enzyme-2 (ACE2); this is followed by stimulation of the cellular protease—Transmembrane Serine Protease 2 (TMPRSS2)—resulting in the fusion of the cell membrane with virus particles [8,9,10]. Olfactory epithelial cells differentially express ACE2 and TMPRSS2. These proteins are present at low levels in neural cells but are present in non-neural cells such as the supporting cells, perivascular cells, or stem cells. No ACE2 expression has been found in olfactory bulb neurons. ACE2 expression has been found in various cell types present in the central nervous system, such as Purkinje neurons, astrocytes, or oligodendrocytes. Some of these cells coexpress TMPRSS2 [8,10]. The mechanism of GD associated with COVID-19 has not been thoroughly investigated, but studies point to two possible mechanisms. One is related to the presence of ACE2 on the mucous membranes of the oral cavity, especially the tongue [11]. The second is related to the fact that the perception of taste depends not only on the taste of the food consumed but also on its smell and temperature. Patients with retronasal OD report accompanying GDs [12,13].

Persistent OD and/or GD (OGD) are common symptoms of post-COVID-19 syndrome. They are a significant problem for those afflicted, as they cause psychological distress and reduce quality of life [14,15].

## 2. Materials and Methods

This paper reports on the results of a prospective, single-center study. The study protocol, consent forms, and patient brochure were approved by the Bioethics Committee at the Institute of Physiology and Pathology of Hearing (number KB.IFPS 7/2021) and were in accordance with the World Medical Association Declaration of Helsinki. Participation in the study was voluntary and cost-free.

### 2.1. Measures

Patients, after consenting to participate in the study, underwent a subjective and physical examination, including nasal endoscopy. Subjective olfactory and gustatory tests were then performed.

### 2.2. Sniffin’ Sticks Test (SST)

The SST is currently one of the most common tests used in the diagnosis of ODs. It was developed in 1997 by Hummel and colleagues [16]. A Polish version of the test was published in 2014 [17]. The test uses absorbent sticks soaked in liquid fragrances dissolved in a solution of propylene glycol. Clinically, the tip of the stick is held about 2 cm from the patient’s nostrils for 3 s. The test has three parts: a threshold test, a discrimination test, and an identification test.

The threshold test is based on 16 sets of sticks. Each set contains two unscented sticks and one stick soaked in an *n*-butanol or 2-phenylethanol (PEG) solution. The examiner presents the patient with triplets of increasing odor concentration. The patient’s task is to indicate which stick carries the odor.

The discrimination test consists of 16 sets of three sticks. Each set contains two sticks with the same odor and one stick with a different odor. The patient’s task is to indicate the stick with the different fragrance.

The identification test consists of 16 sticks, each with a different odor. After an odor is presented, the patient is asked to name the odor with the help of four prompts.

A maximum of 16 points can be scored in each section of the test. After all parts of the test have been completed, the scores are added up. A maximum of 48 points can be obtained. A score above 30 indicates normosmia, 15 to 30 indicates hyposmia, and below 16 is anosmia [18]. The exact rules for the test, the norm ranges, and a centile grid are available with the packaging.

### 2.3. Taste Strips Test (TS)

TS is a psychophysical test to evaluate the sense of taste, published in 2009 by Landis [19]. It consists of a set of impregnated paper strips soaked in substances with four basic tastes (sweet, salty, bitter, and sour) in different concentrations. To perform the test, the investigator places a strip on the patient’s tongue. The patient identifies the taste by naming one of the four possibilities. The manufacturer of the test also offers an abbreviated version consisting of strips with the highest concentration of the taste substances. In our study, we used the short version.

### 2.4. Subjects

The study group consisted of 81 subjects (16 men and 65 women) aged between 12 and 73 years; the mean age was 41.9 (SD = 14.1). All of them presented to the Institute of Physiology and Pathology of Hearing for subjective OD associated with COVID-19.

### 2.5. Inclusion Criteria

The main inclusion criterion for the study was the presence of OD that started during COVID-19 and lasted at least 1 month after the acute symptoms of the disease had subsided. Only patients whose COVID-19 was confirmed by a positive test for the presence of the SARS-CoV-2 virus were included.

### 2.6. Exclusion Criteria

**Figure 1:**
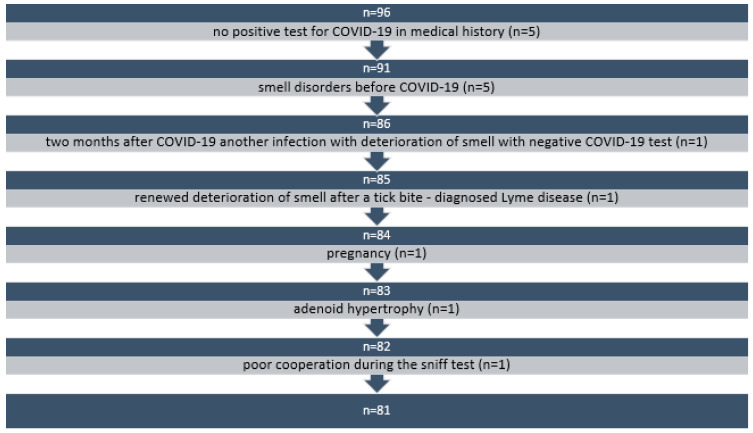
The patient exclusion process.

Patients who did not have a positive COVID-19 test result at the time of the OD were excluded. Those whose OD existed before COVID-19 (and became worse during the disease) were also excluded. We also excluded patients who developed other diseases following COVID-19 that caused deterioration of the sense of smell. Similarly, patients with conditions or diseases that might affect normal olfactory function were excluded (based on medical history and physical examination). A final exclusion criterion was poor cooperation during the tests, which would reduce reliability. A patient exclusion flowchart is illustrated in Figure 1.

### 2.7. Statistical Analysis

Descriptive statistics were used to describe olfactory and gustatory function levels. To compare olfactory function between men and women, a Mann–Whitney *U*-test was used. To test the differences between age groups, a Kruskal−Wallis was used. The relationship between olfactory and gustatory sensitivity was assessed with Spearman-rho correlations. Statistical significance was set at a *p*-value less than 0.05. Analysis was performed using IBM SPSS Statistics v. 24.

## 3. Results

**Figure 2:**
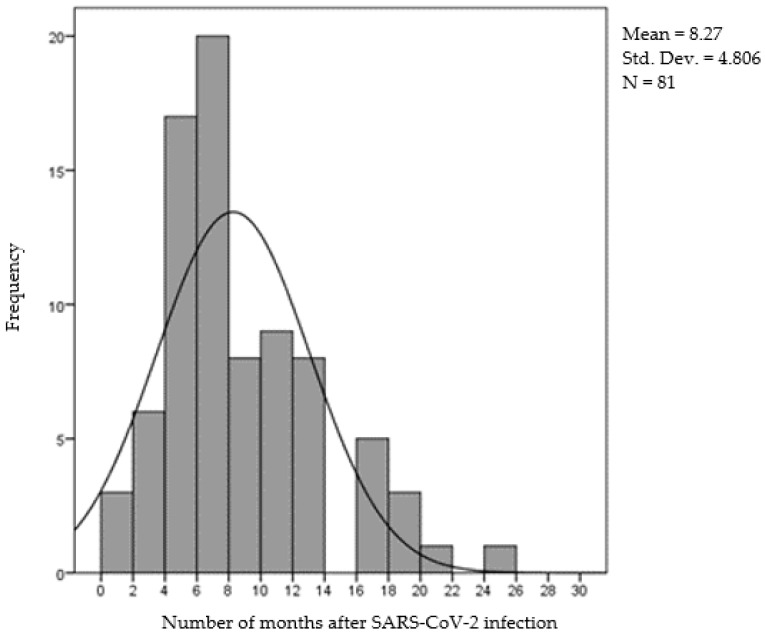
Distribution of the number of months after SARS-CoV-2 infection.

Participants differed in terms of how long they had suffered SARS-CoV-2 infection. The number of months since infection varied from 1 to 25, with a mean of 8.3 months (SD = 4.8). The distribution of months since infection is shown in Figure 2, which shows that most participants had caught the infection less than 8 months prior to the study.

The scores shown in Table 1 are rather low, particularly for the odor threshold. When the total scores (final column) were compared to the standards proposed by Hummel et al. [18], it was found that anosmia (≤15 points) was present in 18 participants (22%), hyposmia (16–30 points) in 52 (64%), and normosmia (>30 points) in 11 participants (14%).

There were 29 subjects (36%) who reported they were currently experiencing phantosmia (odorant is perceived without concurrent stimulus, an imaginary smell). Most often, they perceived the smell of cigarette smoke, an indefinite unpleasant odor, or a burning smell when there were no such materials around. The presence of phantosmia did not correlate with impaired olfactory sensitivity: subjects with phantosmia did not differ from those without in terms of odor thresholds (*U* = 638.5, *p* = 0.224), odor discrimination (*U* = 721.0, *p* = 0.744), odor identification (*U* = 677.5, *p* = 0.448), or total score (*U* = 720.5, *p* = 0.741).

In terms of the identification part of the SST, the subject responses are summarized in Table 2. In general, odors were recognized quite well, but the smell of apples was poorly recognized, with only 14% of patients identifying it correctly.

Detailed results obtained from women and men of different ages are set out in Table 3. There were no statistically significant differences between women and men in terms of olfactory sensitivity as measured with the Sniffin’ Sticks test. The Mann–Whitney *U*-test for odor thresholds was *U* = 496.5, *p* = 0.775; for odor discrimination, it was *U* = 501.5, *p* = 0.825; for odor identification, it was *U* = 449.5, *p* = 0.400; and for the total score, it was *U* = 458.0, *p* = 0.462.

The study group was divided into age subgroups in the same way as carried out by other authors [17,18], i.e., up to 35 years (*n* = 24), 36–55 years (*n* = 42), and over 55 years (*n* = 15). No statistically significant differences were found. The Kruskal–Wallis test for odor thresholds was *H* = 3.04, *p* = 0.219; for odor discrimination, it was *H* = 1.39, *p* = 0.500; for odor identification, it was *H* = 1.37, *p* = 0.503; and for the total score, it was *H* = 1.03, *p* = 0.598.

### 3.1. Taste

In the TS test, the scores varied between 0 and 4 points, with a mean of 3.53 and a standard deviation of 0.91. Most participants (*n* = 59; 73%) obtained the maximum possible score of 4 points; 12 participants (15%) scored 3 points, 5 (6%) scored 2 points, 4 (5%) scored 1 point, and 1 person scored 0 points.

The sweet taste was the easiest to recognize—96% of the participants answered correctly—while the most difficult to identify was salty (84% correct responses). The details are shown in Table 3.

### 3.2. Relationship between Olfactory and Gustatory Sensitivity

There were weak and statistically non-significant correlations between scores obtained in the Sniffin’ Sticks test and the Taste Strips test. The correlations were as follows: for odor thresholds, *rho* = 0.11, *p* = 0.318; for odor discrimination, *rho* = 0.21, *p* = 0.061; for odor identification, *rho* = 0.02, *p* = 0.839; and for total score, *rho* = 0.16, *p* = 0.165.

Results for olfactory sensitivity and taste recognition were compared (Table 4). A sweet taste was always recognized very well, irrespective of the level of olfactory sensitivity (i.e., by all the patients with normosmia and all the patients with anosmia). Generally, equal identification scores were obtained for salty and sour tastes. Only for the bitter taste was there some appreciable difference in that bitter was recognized somewhat poorly by subjects with anosmia, better by those with hyposmia, and best by those with normosmia. However, when the relationships were assessed with a *χ*^2^ test, there was no statistical significance.

## 4. Discussion

One of the most common causes of OD in adults (about 40% of cases) is viral infections [20,21]. ODs and GDs associated with COVID-19 usually resolve spontaneously. However, there is a group of patients in whom ODs and GDs persist despite treatment. In our study, we relied on the results of the SST and TS and the subjective assessment of the presence of phantosmia. Patients with OGDs with durations ranging from 1 to 25 months presented to our clinic. The long time before patients presented may be related to the fact that relatively few medical centers offer help to patients with post-COVID-19 ODs, and knowledge regarding post-COVID-19 ODs is still developing. Some patients began to seek OD treatment after other, more serious complications from COVID-19 had resolved. The varying duration of ODs may depend on the degree of damage associated with SARS-CoV-2 virus penetration. In the case of conductive disorders, olfactory improvement occurs after the acute inflammation of the nasal mucosa has subsided, which explains the improvement in olfactory function in the majority of COVID-19 survivors. In patients with damage to the olfactory cells, ODs persist much longer due to the longer recovery time of the nerve cells and the need to reestablish adequate intercellular connections [7].

The majority of our participants (64%) had hyposmia, while 22% had functional anosmia. The other 14% were within the normal range. Comparing the different parts of the SST showed that subjects scored lowest on the threshold part of the test. The results of the other parts of the test (discrimination and identification) were significantly better, suggesting that if the stimulus is intense enough, incorrect discrimination and identification of odors are less frequent. Similar results have been obtained by other authors, both in patients shortly after a positive COVID-19 test result and in those with ODs lasting more than 4 weeks [6,14,22,23,24,25,26]. In all studies in which the SST was used to assess OD, the threshold test had the most significant effect on the overall test score. The score obtained in this part of the SST depends mainly on the function of the olfactory epithelium, which may suggest that patients with post-COVID-19 OD have mainly peripheral olfactory damage as a result of a cytokine storm, causing leukocytic infiltration and damage to olfactory epithelial cells, including stem cells [23]. It should be noted that the threshold test is a relatively difficult test to perform due to the execution procedure and longer digestion time than the other SST tests. In the Polish population, even in patients with normosmia, the score obtained in the identification test is lower than in the other parts of the SST, whereas the difference is much smaller than in the post-COVID OD patients included in our study [17].

The results showed that recognition of the specific odors used in the identification part of the test was quite good, with the exception of the apple odor stick. This may perhaps be due to the fact that the smell of red apples is most familiar to Polish people, whereas the SST uses the smell of green apples [17]. We did not observe differences in recognition between odorants that stimulate just the olfactory nerve and those (such as mint or lemon) that also stimulate the trigeminal nerve.

Some of our patients who reported subjective ODs scored within the normal range on the SST test. Perhaps these patients would have scored higher before they caught COVID-19. Despite a normosmia rating, it is possible they experienced OD and associated discomfort [4]. In the taste tests, an abnormal result was observed in 17% of the subjects. The best-recognized taste was sweet (96%), and the worst was salty (84%). Among the three patient groups (anosmia, hyposmia, and normosmia), sweet was also the best-recognized taste. In all groups, salty and sour tastes were identified by a similar percentage of respondents. Only in the case of bitter was there a slight correlation between TTS and TS scores: it was less well recognized in the anosmic group, while 100% of normosmic subjects recognized it. Initial reports of GD occurring in COVID-19 cases showed a correlation between GD and OD. The results of these studies were often based solely on questionnaire results [5,27]. More recent findings by Prem and colleagues indicate that in patients with short-lasting disorders (up to 4 weeks), TS scores correlate well with information from medical interviews, whereas there is a weak correlation between persistent GD and OD [26,28]. Some publications have found a different prevalence of disorders depending on the taste concerned: for example, in the study by Arndal et al., the least recognized taste was bitter [14], while Singer-Cornelius et al. found it was sour [29].

The relationship between GD and OD remains unclear. Type II G-protein-coupled receptors, like olfactory receptors, are responsible for the perception of sweet, bitter, and umami. Salty and sour are linked to ion receptors (type I) [14]. So far, it has not been possible to demonstrate which receptors are more susceptible to the adverse effects of the SARS-CoV-2 virus. Some patients who have normal taste tests report that they have no problems recognizing basic tastes but have problems identifying different foods. This points to the problem lying more with retronasal olfaction and impaired detection of flavors (which are a mixture of odor and taste compounds), not basic tastes [13,30].

SST scores showed there were no gender-related differences in OD severity. There were more women than men among those presenting to IFPS for post-COVID OD. This may be related to the fact that women experience more psychological distress from illness and are more likely to seek medical help [15,26]. However, our analysis indicated that ODs were not associated with gender, and there was no correlation between age and SST score.

This study has limitations. We obtained information on previous COVID-19 status mainly from a medical interview with the patient—our subjects did not have test results with them during their visit. The initial state of their sense of smell and taste is unknown, as they only presented to our hospital after the onset of ODs. We did not have a taste test kit containing an umami sample; in further studies, consideration should be given to including it in the protocol to allow a broader assessment of GD. Quality of life was also not measured due to the lack of an available Polish version of the QOD (Questionnaire of Olfactory Disorders). Discussions with our patients indicated that the severity of OD did not always correlate with reduced quality of life, and here, an appropriate questionnaire would allow better testing. Further research on post-COVID-19 OD could also be expanded to include objective tests, which were not performed due to a lack of appropriate equipment.

Currently, there is no fully effective treatment for post-COVID OD, and no pharmacological treatment protocol exists. Some patients do experience an improvement in olfactory function after olfactory training, but the limitation here is a lack of immediate results and the need for good cooperation and high patient motivation. Research into other therapies, such as platelet-rich plasma (PRP) injections into the olfactory cleft or direct stimulation of the olfactory bulb, is ongoing [4]. Our subjects were offered the opportunity to participate in a further study to evaluate the effectiveness of olfactory therapies, and the results of this study are being prepared.

## 5. Conclusions

This study looked at the severity and nature of ODs in the Polish population, and its findings are consistent with those from other countries. The data may contribute to a better understanding of ODs associated with SARS-CoV-2 virus infection. Our tentative conclusion is that ODs associated with COVID-19 are probably more peripheral than central. This suggests that, when physically examining a patient with OD, clinicians should pay particular attention to the condition of the nasal mucosa and instruct the patient on the basic principles of good nasal hygiene and the environmental factors that can affect the function of the olfactory epithelium.

We estimate there may be several million people worldwide experiencing persistent OD associated with COVID-19. The pathophysiological mechanisms behind OD are increasingly being understood. Research into the causes and nature of OD is important because it provides a starting point in the search for effective OD treatments.

## Figures and Tables

**Table 1:** Descriptive statistics for Sniffin’ Sticks test scores obtained by 81 subjects with olfactory dysfunction after COVID-19.

	Odor Threshold	Odor Discrimination	Odor Identification	Total Score
N	81	81	81	81
M ± SD	3.34 ± 4.72	9.89 ± 3.10	9.12 ± 3.00	22.35 ± 7.78
Min	0.00	0.00	2.00	3.00
Max	16.00	16.00	15.00	40.00
P10	0.00	6.00	5.00	11.20
P25	0.00	8.00	7.00	18.00
P50	1.00	10.00	9.00	23.00
P75	4.00	12.00	11.00	27.00
P90	14.50	13.00	13.00	32.00

N, number of subjects; M, mean, SD, standard deviation; Min, minimum; Max, maximum; P10, 10th percentile; P25, 25th percentile; P50, 50th percentile; P75, 75th percentile; P90, 90th percentile.

**Table 2:** Answers provided by the subjects in the identification section of the SST.

Task	Correct Answer	Incorrect Answers			No Answer
1	Orange, 88.9%	Blackberry, 3.7%	Strawberry, 2.5%	Pineapple, 4.9%	-
2	Leather, 33.3%	Smoke, 24.7%	Glue, 23.5%	Grass, 13.6%	4.9%
3	Cinnamon, 53.1%	Honey, 12.3%	Vanilla. 21%	Chocolate, 7.4%	6.2%
4	Mint, 65.4%	Chives, 4.9%	Fir, 23.5%	Onion, 4.9%	1.2%
5	Banana, 59.3%	Cocoa, 13.6%	Walnut, 9.9%	Cherry, 13.6%	3.7%
6	Lemon, 43.2	Peach, 3.7%	Apple, 3.7%	Grapefruit, 43.2%	3.7%
7	Licorice, 42%	Cherry, 12.3%	Mint, 9.9%	Cookies, 32.1%	3.7%
8	Turpentine, 38.3%	Mustard, 4.9%	Gum, 9.9%	Menthol, 44.4%	2.5%
9	Garlic, 49.4%	Onion, 28.4%	Sauerkraut, 19.8%	Carrot, 2.5%	-
10	Coffee, 80.2%	Cigarettes, 8.6%	Wine, 2.5%	Smoke, 6.2%	2.5%
11	Apple, 13.6%	Melon, 53.1%	Peach, 21%	Orange, 9.9%	2.5%
12	Cloves, 76.5%	Pepper, 4.9%	Cinnamon, 17.3%	Mustard, 1.2%	-
13	Pineapple, 51.9%	Pear, 21%	Plum, 6.2%	Peach, 17.3%	3.7%
14	Rose, 70.4%	Chamomile, 14.8%	Raspberry, 3.7%	Cherry, 6.2%	4.9%
15	Aniseed, 56.8%	Rum, 9.9%	Honey, 9.9%	Fir, 18.5%	4.9%
16	Fish, 86.4%	Bread, 6.2%	Cheese, 2.5%	Ham, 3.7%	1.2%

**Table 3:** Results of the taste test.

	Correct	Incorrect
Sweet	78 (96.3%)	3 (3.7%)
Salty	68 (84%)	13 (16%)
Sour	72 (88.9%)	9 (11.1%)
Bitter	70 (86.4%)	11 (13.6%)

**Table 4:** Level of olfactory sensitivity and correct recognition of tastes.

	Sweet	Salty	Sour	Bitter
Anosmia (*n* = 18)	100%	83.3%	88.9%	77.8%
Hyposmia (*n* = 52)	94.2%	80.8%	88.5%	86.5%
Normosmia (*n* = 11)	100%	84.0%	90.9%	100%

## Data Availability

The data that support the findings of this study are available from the corresponding author [P.H.S.] upon reasonable request.
